# Do the core symptoms play key roles in the development of problematic smartphone use symptoms

**DOI:** 10.3389/fpsyt.2022.959103

**Published:** 2022-09-06

**Authors:** Shunsen Huang, Xiaoxiong Lai, Yajun Li, Xinran Dai, Wenrong Wang, Jing Li, Huanlei Wang, Dufang Li, Yun Wang

**Affiliations:** ^1^State Key Laboratory of Cognitive Neuroscience and Learning, Beijing Normal University, Beijing, China; ^2^Guangming Institute of Education Sciences, Shenzhen, China; ^3^Zhongmu Education Teaching and Research Office, Zhengzhou, China; ^4^Jiyuan Gaoji Zhongxue, Jiyuan, China; ^5^Experimental Primary School, Experimental Primary School of Beijing Normal University, Beijing, China

**Keywords:** problematic smartphone use, network analysis, core symptom, cross-lagged panel model, graphical vector autoregression model

## Abstract

**Aims:**

Previous research determined the core symptoms (loss of control and being caught in the loop) of problematic smartphone use (PSU), which are of great importance to understand the structure and potential intervention targets of PSU. However, the cross-sectional design fails to reveal causality between symptoms and usually conflates the between- and within-subjects effects of PSU symptoms. This study aims to determine whether the core symptoms of PSU, indeed, dominate the future development of PSU symptoms from longitudinal between- and within-subjects levels.

**Materials and methods:**

In this study, 2191 adolescents were surveyed for 3 years for PSU symptoms. A cross-lagged panel model (CLPM) was used to explore longitudinal between-subjects causal relationships between symptoms, and a graphic vector autoregressive model (GVAR) was used to separate the between- and within-subjects effects and detect the longitudinal effect at the within-subject level.

**Results:**

The results of CLPM indicated that the core symptoms (both loss of control and being caught in the loop) of PSU, indeed, dominate the future development of PSU symptoms at a longitudinal between-subjects level. From T1 to T2, the cross-lagged model showed that both the loss of control (out-prediction = 0.042) and being caught in the loop (out-prediction = 0.053) at T1 have the highest out-prediction over other symptoms at T2. From T2 to T3, the loss of control (out-prediction = 0.027) and being caught in the loop (out-prediction = 0.037) at T2 also have the highest out-prediction over other symptoms of PSU at T3. While, after separating the between- and within-subjects effects, only being caught in the loop at T1 played a key role in promoting the development of other PSU symptoms at T3 at the within-subjects level. The contemporaneous network showed intensive connection, while the cross-sectional between-subjects network is very sparse.

**Conclusion:**

These findings not only confirm and extend the key roles of core symptoms in the dynamic aspect of PSU symptoms and PSU itself but also suggest that interventions should consider the core symptoms of PSU, individual- and group-level effects and that individualized intervention programs are needed in future.

## Introduction

The popularity of smartphones has brought great convenience to people, such as online shopping, online payments, online reading, and online communication. Although a growing number of studies have recently provided evidence revealing that smartphone use is negatively related to adolescents’ future mental health or wellbeing (1, 2), there is no doubt that smartphone overuse may develop into problematic smartphone use (PSU), which is regarded as the inability to regulate or control one’s use of smartphones, eventually involving negative outcomes in daily life (3–5). There is no consensus on the criteria related to identifying smartphone use as an addictive behavior or *Use Disorder*. Montag et al. (6) argued that it is important to consider both the type of internet content and device differences in the classification of internet use disorders (IUDs). Namely, specific internet use motivations and needs may trigger preference for specific types of internet content, and different devices have specific behavioral use patterns, technical characteristics, or preferences for a particular type of content (6). Thus, researchers proposed to divide IUD into smartphone and non-smartphone IUD, and researchers suggested that PSU (or smartphone use disorder) can be defined as pervasive, unspecified IUD, predominantly mobile *via* a smartphone (6, 7). Systematic meta-analysis and longitudinal studies have shown that PSU is associated with increased depression, anxiety, perceived stress, and poor sleep quality (8–11). Researchers argued that it is time to be prepared to address technological addictions in psychiatric practice (12).

Researchers have recently focused on the internal structure of PSU, i.e., studying the interconnections between PSU symptoms from a network perspective, which has contributed to the understanding of PSU network structure and the potential values of interventions regarding PSU (4, 13–15). The network analysis is based on the network theory of mental disorders (16–18), suggesting that causal interactions between symptoms can automatically produce feedback features of mental disorders. Thus, from this causal relationship between symptoms, an intervention to change the status of one symptom would change the probability distribution of other symptoms. This indicates that the symptoms with the greatest impact on the network should be prioritized in an intervention (16, 19). The network analysis method visualizes the interactions between symptoms, where nodes represent symptoms and edges between nodes represent directed (e.g., cross-lagged panel model, CLPM) or undirected (e.g., graphic gaussian model, GGM) relationships between them (20, 21).

When exploring the interconnections between symptoms from a network perspective, Huang et al. (4) found that loss of control and continued excessive smartphone use were core symptoms of PSU in a cross-sectional sample of students. Andrade et al. (13) also indicated that loss of control had the highest centrality in both students and adults samples. Network analysis on internet addiction revealed similar results that spending more time online is regarded as the most core symptom of internet addiction (22) and deficient self-regulation is the most core symptom of problematic social media use (23). Except for the core symptoms of PSU, some researchers have begun to focus on the relationships between inner PSU symptoms and outer various elements like personality and family environments. For example, Wei et al. (24) examined the relationship between neuroticism and PSU symptoms, Li et al. (25) explored the relationships between fear of missing out, PSU symptoms, and symptoms of problematic social network site use, and Huang et al. (15) explored the relationships between PSU symptoms and their influences such as personal characteristics, family environment, and school environment. Based on these, researchers argued that intervention focusing on these core symptoms or components of PSU and related core influences would be effective and economic (4, 15, 24, 25).

Although previous literature has clarified the core symptoms of PSU, they are based on cross-sectional data that fail to determine causality between symptoms, which prevent researchers from knowing whether focusing on these core symptoms, indeed, relieves PSU or PSU symptoms in future. Thus, it is essential to examine the longitudinal causal development of PSU symptoms to determine whether the core symptoms of PSU, indeed, dominate the development of other symptoms. Besides, existing studies using network analysis have been unable to distinguish the longitudinal between- and within-subjects effects of PSU symptoms. Researchers have argued that the within- and between-subjects levels may yield different results and theories, which are often misunderstood by researchers [e.g., many theories generated from the within-subjects level were evaluated from the between-subjects level by using cross-sectional data (26)]. Within-subjects effects represent the inner variation of the participants (e.g., when individuals score higher than usual on symptom A, they experience a subsequent increase in symptom B), while the between-subjects effect represents the variation between participants (e.g., when individuals score low on symptom A, they experience a subsequent decreasing rank of symptom B, compared to individuals who score high on symptom A). Researchers have suggested that the between-subjects effect reveals the influence of culture, policies, or social atmosphere (26). Therefore, when examining the causality between symptoms, we need to explore whether core symptoms play dominant roles in the development of PSU symptoms from the perspective of both between- and within-subjects effects.

Researchers have developed CLPM and graphical vector autoregression (GVAR) model to detect longitudinal relationships between symptoms. CLPM allows for the study of examining the longitudinal processes of disorders by characterizing the effect of observed symptoms on each other over time (27). This model would allow for individual symptoms of a disorder to affect other symptoms over time. This model is typically used to detect a longitudinal between-subjects effect and is only appropriate for data with two-time points. The GVAR (28) processes data with at least three-time points and can separate between-subjects and within-subjects effects and form the temporal network, contemporaneous network, and stationary between-subjects network. The between-subjects network is an undirected GGM between the means of scores of different subjects under different measurements. The contemporaneous network is also an undirected GGM within the same measurement. The temporal network is a directed network that indicates within-person variation across time, and its relationships are usually directed and predictive regression coefficients.

Although GVAR extracts the within-subjects effects and between-subjects effects, the between-subjects network is cross-sectional (20) and fails to detect sustained prospective effects (e.g., longitudinal between-subjects effect) (29) because GVAR only captures temporal fluctuations around individual means and ignores long-term effects that persist over time (30). As previous studies have shown that CLPM facilitates the prospective understanding of between-subjects effects, though it may mix with within-subjects effects, it is still recommended and used by many researchers because it can detect the longitudinal and consistent between-subjects effect across different samples (29). Due to the limitations and strengths of CLPM and GVAR, CLPM must be conducted to obtain the longitudinal between-subjects effect of PSU symptoms and GVAR should be used to obtain the longitudinal within-subject effect (temporal network). In this way, our understanding of the nature of PSU symptom development will be enriched and different suggestions for PSU interventions can be disclosed from multiple perspectives (26–28, 31).

Regarding the limitations of previous cross-sectional networks of PSU symptoms, this study uses three waves of PSU data to explore the causality between PSU symptoms. A recent empirical study used qualitative and quantitative methods in a real-life context to examine how smartphone interactions are driven by a complex set of routines and habits that users develop over time (32). They found that users would get caught in the loop when they engage with their smartphones for a longer period of time (32), which is consistent with the symptom of continued excessive smartphone use behavior. Thus, in the next analysis, we used the term “being caught in the loop” to represent continued excessive smartphone use behavior. In CLPM, we assumed that core symptoms (loss of control and being caught in the loop) play a key role in the dynamic development of PSU symptoms. Besides, in GVAR, we assumed that the core symptoms also play key roles in the within-subjects network, whereas the between-subjects network may differ from formers.

## Materials and methods

### Participants

Two thousand one hundred ninety-one participants, from 55 classes of 12 randomly selected primary and secondary schools in Henan province, were surveyed at three-time points (April 2019, July 2020, and April 2021). We randomly selected these schools from a list of schools downloaded from the local government’s website and then contacted the schools and obtained their permission to conduct this study. In wave 1, 2191 (2123 are valid) participants were surveyed and completed a paper-and-pencil questionnaire. In wave 2, 2191 (2191 are valid) participants were surveyed online due to the onset of COVID-19. In wave 3, 2191 (2188 are valid) participants were surveyed again and completed the paper-and-pencil questionnaire. Teachers helped collect the completed questionnaires and sent online survey links to the students. Participants with unanswered questionnaires in any wave were excluded, leaving 2118 participants (male = 1027, average age = 12.16 ± 2.28). Little’s MCAR test was used to examine the types of missing data retained (mean missing = 0.43%), and the results show that the data were not MCAR (χ^2^ = 556.314, df = 4531, *p* < 0.001), and the EM algorithm imputation method was used to process the missing data (33). The demographic information of the participants is shown in Table 1. This study was approved by the Institutional Review Board (IRB) of the State Key Laboratory of Cognitive Neuroscience and Learning, Beijing Normal University. And the informed consent from parents and children were obtained appropriately.

**TABLE 1:** Demographic information and descriptive statistics.

Variables	Groups	Percentage (%)	Waves	PSU mean (SD)	*t*-test
Residence	City	47%	T1	1.864 (0.61)	
	Township	15.3%			T2 > T1 (*t* = −5.369)**
	Rural region	37.7%			
Only child	Yes	91%			
	No	9%			
Mother’s education	<College	90.5%	T2	1.950 (0.66)	T3 > T1 (*t* = −5.579)**
	≥College	9.5%			
Father’s education	<College	87.5%			No difference between T2 and T3 (*t* = 0.307)
	≥College	11.4%			
Annual income	<50,000¥	60.2%	T3	1.945 (0.64)	
	50,000¥–100,000¥	21.6%			
	>100,000¥	19.2%			

¥ = RMB. **p < 0.001. The mean (SD) of PSU was calculated based on items used in Table 2.

### Measurement

The Smartphone Addiction Proneness Scale for Youth (SAPS-Y) revised to be suitable for Chinese adolescents (4) was used. The revised scale consists of 16 items (four-point Likert scale) and contains four dimensions: (1) disturbance of adaptive functions; (2) withdrawal; (3) tolerance; and (4) virtual life orientation. A higher score means a higher PSU.

In network analysis, researchers usually renamed each item with a specific symptom (4, 15, 23). It is important to reduce redundancy in the network since redundancy may lead to inaccurate estimates (27, 34). In previous PSU networks, redundancy may exist between symptoms (4). The literature provides two ways to reduce redundant items, one relies on theory (27) and the other is with the help of redundancy analysis software (e.g., *EGAnet* package). In this study, three items from the dimension of virtual life orientation and one item revealing boredness were removed. There are several reasons for this practice. First, these items are not mentioned in the internet gaming disorder (IGD) in the DSM-5 and the gaming disorder in ICD-11. The IGD criteria were used because a recent research on the taxonomical issue of IUD considered PSU to be a generalized, unspecified IUD, predominantly mobile *via* a smartphone. They argue that gaming disorder can also be a specified IUD, predominantly mobile *via* a smartphone (6). Besides, many researchers have also developed PSU scales using the IGD criteria [see a review by Harris et al. (35)]. Second, the items dropped from the dimension of virtual life orientation were not extremely relevant items for PSU (36). Kwon et al. (36) used an experts-rating method to select 10 items (symptoms) that were most relevant for representing PSU and conducted a rigorous procedure to test the reliability and validity of the one-dimension scale. The most relevant symptoms involved interference with planned work and life, being hard to concentrate, withdrawal symptoms like impatience and fretfulness, loss of control, and excessive use (36). Third, recent research has found that the dimension—virtual life orientation in SAPS-Y was problematic in Chinese samples, and only one item of this dimension was retained in a sample of Chinese adolescents (37). Besides, the number of nodes in the network should be small because a large number of nodes may reduce the sensitivity of network estimation and accuracy of GVAR (38). Thus, the four items were dropped in network analysis. Then, according to redundancy analysis, four pairs of two items expressing the same meaning were combined into one item by averaging, because this approach allows more information to be retained than selecting one of the items (27). The items and corresponding abbreviations are shown in Table 2 in the Appendix.

**TABLE 2:** Detailed information about PSU symptoms and related references.

Items	Meanings and related references	Abbreviation
I have a hard time doing what I have planned (study, do homework, or go to afterschool classes) due to using a smartphone	Jeopardizing education or relationships [Criterion 1 of IGD; (4, 36, 46)]	Jeopardization
Family or friends complain that I use my smartphone too much		
I get anxious and restless when I am without a smartphone by my side	Withdrawal symptoms are experienced when internet gaming is taken away [Criterion 2 of IGD; (4, 36, 46, 52)]	Withdrawal
I feel nervous if I couldn’t check my smartphone or open my smartphone		
I cannot imagine life without a smartphone	Internet gaming becomes the dominant activity (Criterion 1 of IGD; (4, 46))	Preoccupation
I use a smartphone to make me feel better when in a bad mood	Escaping or relieving a negative mood [Criterion 8 of IGD; (46)]	Alleviation
I try cutting my smartphone use time, but I fail	Unsuccessful attempts at self-control [Criterion 4 of IGD; (4, 36, 52)]	Loss of control
Even when I know I should stop, I continue to use my smartphone too much		
Using a smartphone is more enjoyable than spending time with family or friends	Loss of interest activities except for internet gaming [Criterion 5 of IGD; (46)]	Loss of interests
I find that the time I spend on my smartphone is longer than planned.	The need to spend increasing lengths of time engaged in internet games [Criterion 3 of IGD; (32, 36, 46)]	Being caught in the loop
Spending a lot of time on my smartphone has become a habit		
My smartphone does distract me from what I am doing.	The distraction caused by smartphone use (4, 36)	Distraction

Taken into account, items rather than dimensions were used as symptoms and that the eight symptoms were similar to the most relevant symptoms in a one-dimension PSU scale (36). We calculated reliability and validity for the eight symptoms in the SAPS-Y, indicating good reliability (α_*wave*1_ = 0.827, α_*wave*2_ = 0.923, α_*wave*3_ = 0.887) and construct validity (CFA_*wave*1_:χ^2^ = 136.504, *df* = 17, *p* < 0.001, CFI = 0.977, TLI = 0.968, RMSEA = 0.058; CFA_*wave*2_:χ^2^ = 239.249, *df* = 17, *p* < 0.001, CFI = 0.981, TLI = 0.969, RMSEA = 0.079; CFA_*wave*3_:χ^2^ = 161.138, *df* = 17, *p* < 0.001, CFI = 0.983, TLI = 0.973, RMSEA = 0.063).

### Analytic procedure

First, SPSS 25.0 was used to handle the missing values, demographic information, and descriptive results of PSU. Second, the redundant items of PSU were removed or merged according to theory (relying mainly on IGD in DSM-5) and redundancy analysis (using R package *EGAnet*, (34)) before performing network analysis. Third, we used CLPM to construct two models presenting cross-lagged effects between symptoms from T1 to T2 and T2 to T3. The in-prediction and out-prediction of each symptom on other symptoms were calculated as centrality, with higher in-prediction of a symptom implying that the symptom is more likely to be influenced by other symptoms, and higher out-prediction. Fourth, to separate the between- and within-subjects effects, the GVAR model was applied using the R package *Psychonetrics* (39). In GVAR, temporal dependencies are regressed on the previous measurement occasion to obtain a directed network—a temporal network. The variances and covariances remaining after controlling for temporal effects can be modeled as a contemporaneous network. The temporal and contemporaneous are obtained by average. When a group of subjects is modeled, between-subjects effects can form a between-subjects network by averaging the long-term effects across people. In this study, the GVAR model showed a good fit [NFI = 0.93, TLI = 0.93, NNFI = 0.93, RFI = 0.92, IFI = 0.94, RNI = 0.94, CFI = 0.94, RMSEA = 0.056 (95% CI:0.053,0.058)] (20). Besides, to determine the centrality of symptoms in the GVAR, strength (the absolute value of the weights on the edges connected to a node) and outstrength (the most predictive value toward other nodes at the next time point) were used. Outstrength was used for the temporal network, and strength was calculated for contemporaneous and between-subjects networks.

## Results

### Demographic information and descriptive statistics of problematic smartphone use

Table 1 shows that the mean score of PSU increases from T1 to T2 and then quits at T3. Further *t*-test shows no difference in PSU score at T2 and T3.

### Results from the cross-lagged model

**FIGURE 1:**
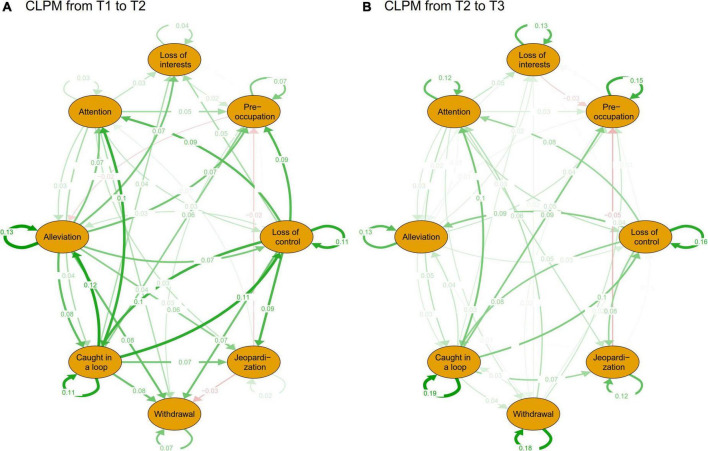
The CLPM of PSU of T1 to T2 and T2 to T3. **(A)** Is the CLPM from time 1 to time 2 and **(B)** is the CLPM from time 2 to time 3. CLPM is a cross-lagged panel model. Green paths represent positive prediction and red path means negative prediction. For comparison, the layout is set to “circle.” The node paths directed to themselves are autoregressive paths and the rest are cross-lagged paths.

**FIGURE 2:**
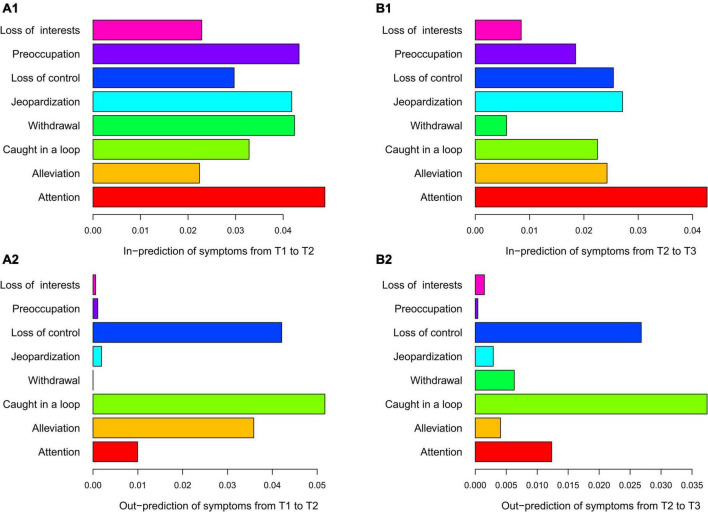
The centrality of the CLPM of PSU. **(A1)** is in-prediction of symptoms from T1 to T2, **(A2)** is out-prediction of symptoms from T1 to T2, **(B1)** is in-prediction of symptoms from T2 to T3, and **(B2)** is out-prediction of symptoms from T2 to T3. CLPM is the cross-lagged panel model. The in- and out-predictions were summed by the squared regression paths from one symptom to another. According to the suggestion of Rhemtulla et al. (27), the autoregressive path was excluded when calculating prediction to highlight the cross-lagged effect.

In Figure 1, CLPM symptoms from T1 to T2 and T2 to T3 are mutually predicted, with the former network showing more intensive prediction paths than the latter. Most of the symptoms at the former time positively predict the symptoms at the next time, with only a few weak and negative predictions (β = −0.02 to −0.05) in both networks. Combining the information in Figure 2, the highest in-prediction symptoms are attention in CLPM from T1 to T2 (Figure 2A1) and from T2 to T3 (Figure 2B1). In contrast, the highest out-prediction symptoms are being caught in the loop and loss of control from T1 to T2 (Figure 2A2) and from T2 to T3 (Figure 2B2). Besides, the out-prediction of being caught in the loop and loss of control is much lower from T1 to T2 than from T2 to T3. Considering the PSU score in Table 1, when the PSU score increases from T1 to T2, the out-predictions of loss of control (out-prediction = 0.042) and being caught in the loop (out-prediction = 0.053) are high, while the PSU score stops increasing from T2 to T3, the out-predictions of the loss of control (out-prediction = 0.027) and being caught in the loop (out-prediction = 0.037) also decrease (see Figure 2). This indicates that both the core PSU symptoms and the PSU score have a similar developmental pattern.

### Results from the graphic vector autoregressive model

#### Temporal network

**FIGURE 3:**
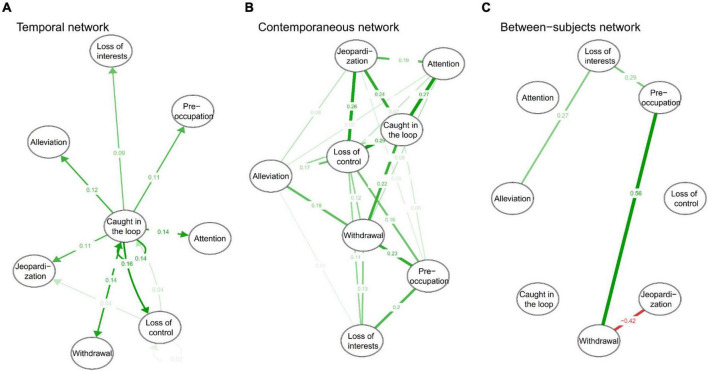
Within- and between-subjects networks of PSU symptoms. **(A)** is temporal network, **(B)** is contemporaneous network, and **(C)** is between-subjects network. The green path represents positive prediction or correlation and the red path means negative prediction or correlation. The node paths directed to themselves (in the temporal network) are autoregressive paths, and the rest are the cross-lagged paths at the within-subjects level.

**FIGURE 4:**
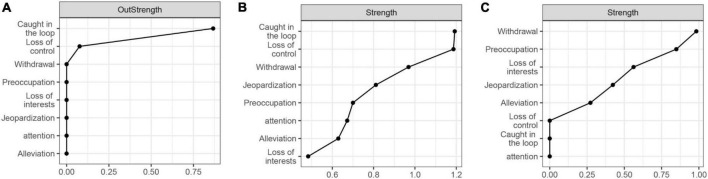
Centrality of within- and between-subjects networks. **(A)** is the centrality of the temporal network, **(B)** is the contemporaneous network, and **(C)** is the between-subjects network.

Figure 3A shows that being caught in the loop positively predicted the other seven symptoms in future. Of the eight symptoms, only loss of control and being caught in the loop were mutually predictive, with loss of control positively predicting later being caught in the loop (β = 0.04). In addition, loss of control predicted the future jeopardization of education or social relationships (β = 0.04). Centrality analysis (Figure 4A) shows that being caught in the loop had the highest centrality (outstrength = 0.866), suggesting that this symptom dominates the overall development of PSU symptoms at the dynamic within-subjects level.

#### Contemporaneous network

Figure 3B presents that adolescents’ PSU symptoms interact with each other with partial correlations ranging from 0.04 to 0.29. The contemporaneous correlations indicate that when individuals exhibit one symptom, they may simultaneously exhibit other symptoms associated with that symptom. The centrality analysis (Figure 4B) shows that the centrality of both being caught in the loop and loss of control was highest in the contemporaneous network (strength = 1.19). This suggests that loss of control and being caught in the loop play extremely important roles in maintaining the contemporaneous PSU symptom network.

#### Between-subjects network

Figure 3C presents the stationary between-subjects network and only four correlations were found. Alleviation is positively correlated with loss of interest (*r* = 0.27), and withdrawal is positively correlated with preoccupation (*r* = 0.56) and negatively correlated with jeopardization (*r* = −0.42). In addition, preoccupation was associated with loss of interest (*r* = 0.29). The between-subjects network was very sparse, unlike the contemporaneous network. Centrality analysis (Figure 4C) reveals that withdrawal had the highest centrality (strength = 0.98).

## Discussion

This longitudinal study explored dynamic aspects of PSU symptoms in adolescents at the within- and between-subjects levels. The results show that core PSU symptoms dominate the development of PSU symptoms at the between-subjects level. In contrast, after separating within- and between-subjects effects of CLPM, only “being caught in the loop” dominates the dynamic development of PSU symptoms at the within-subjects level. Besides, the stationary between-subjects network is extremely different from the networks at the within-subjects level.

In CLPMs, the results support our hypothesis that symptoms are mutually predictive in terms of the longitudinal between-subjects level and those core symptoms in PSU do have a profound effect on other symptoms at later time points. Namely, adolescents with low core symptoms prediction subsequently experience a stepwise decline in other symptoms compared to adolescents with high core symptoms prediction. Previous cross-sectional literature has identified the core role of the loss of control and being caught in the loop (4, 13), and our results extend previous findings by showing their continued critical role in the longitudinal between-subjects level in developing and maintaining the PSU symptom network. Another interesting finding is that the out-prediction of core symptoms is strong as the PSU score increases (T1 to T2) and decreases when the PSU score does not change (T2 to T3). This result may further support that the development of PSU itself may be reflected in the inner change in its core symptoms, which may be of potential value in future intervention programs. In addition, CLPMs show that attention (being distracted by smartphones) is most predicted by other symptoms, which is consistent with previous research that smartphone use increases smartphone interference in life and thus distracts users from their attention and life (40). Intervention strategies proposed by previous cross-sectional network studies (e.g., schools, teachers, and parents should set a time limit on smartphone use to help adolescents avoid being caught in the loop, train children to exercise self-control over their smartphone use, and develop adaptive regulation strategies for smartphones (4)] are further supported in the longitudinal cross-lagged networks.

The longitudinal within-subject network (temporal network) shows that being caught in the loop can affect other PSU symptoms, with little mutual prediction found between being caught in the loop and loss of control, suggesting that only being caught in the loop plays a central role at the within-subjects level in stimulating the formation of PSU symptoms. Namely, adolescents with higher strength of being caught in the loop than usual will experience a subsequent increase in other PSU symptoms. These results are partially consistent with our hypothesis and differ from the results of CLPMs, which we attribute to the separation of within- and between-subjects effects. Besides, the contemporaneous network reveals that both loss of control and being caught in the loop have high centrality, unlike the temporal network where only being caught in the loop has higher centrality. We believe that this is also due to the separation of effects, with the former coming from a longitudinal perspective and the latter from a contemporaneous aspect. Loss of control and being caught in the loop should be regarded as orthogonal dimensions rather than as opposite poles of a single dimension (41), which is supported by the bidirectional relationship between the two symptoms in CLPM and GVAR, although their bidirectional predictive relationship is weak in the temporal network.

The between-subjects network differs from within-subject networks in GVAR. There are two reasons for this, one is that small sample size may lead to a sparse between-subjects network in GVAR (28), but this study is based on a relatively large sample and the other factor concerns a considerable level of variation between-subjects such that the between-subjects network in GVAR could not capture consistent relationships across adolescents. This finding suggests that future treatments should also be mindful of between-subjects differences and focus on personal intervention strategies, which may support the argument used in substance use treatment that a single treatment approach is never sufficient to address substance use disorders due to heterogeneity among patients (42). Therefore, future interventions may attempt to develop individualized training or intervention programs to help alleviate adolescents’ PSU (42, 43).

The results show the difference between the longitudinal between-subjects network (CLPM) and the stationary between-subjects network in GVAR. This difference should be attributed to the separation of temporal and contemporaneous effects, and the between-subjects network in GVAR shows only a stationary effect. Although mixed with the within-subject effect in CLPM, the difference between CLPM and GVAR should not refuse the longitudinal between-subjects effect of CLPM, because it yields a consistent and longitudinal between-subjects effect than other models of separating effects (29). The same is true of the difference between CLPM and temporal networks. As researchers have argued, the cross-lagged effect in CLPM should not be understood as the sum of the within- and between-subjects effects, although the total variance is the sum of them, and the time-varying construct factors have different meanings in different models (29).

In addition, there are few negative predictions and correlations between symptoms in CLPM and GAVR. The negative predictions in CLPM may be because it mixes within- and between-subjects effects. For example, after the effects were separated, negative relationships will still be present in the between-subjects network. Moreover, these negative predictions are very low, which may be related to some form of measurement bias of rarity and spurious effects (44). Future research should focus on these negative predictions and whether they can be replicated. For the strong negative relationship between withdrawal and jeopardization in the between-subjects network in GVAR, we think that it should be explained from the group-level. This relationship may be that high jeopardization may remind adolescents of points of caution, which may cause adolescents to be less addicted to their smartphones and feel less anxiety or restlessness without them. This could also be that once adolescents no longer feel anxious when withdrawing their smartphone use, their jeopardization of education, or social relations increases. The former explanation is more reliable and logical. As suggested by previous literature, the between-subjects level reveals the influence of social culture or government policies (20, 26, 28). Therefore, culture or policies should be considered when explaining this negative association. This negative relationship may be due to government rules regulating students’ smartphone use in school and a social climate that emphasizes the dangers of smartphones. Thus, government policies or school regulations may act to regulate smartphone use among students once their education or social relationships are negatively affected. The Ministry of Education of the People’s Republic of China (MEPRC) recently issued a policy prohibiting primary and secondary school in principle from bringing their smartphones to school (45). This may have a protective effect on adolescents’ PSU, and adolescents may then feel fewer withdrawal symptoms. This may indicate that government policies or school supervision rules regarding smartphone use should also be constantly adopted to reduce withdrawal symptoms in adolescents.

### Limitation

First, there was no consensus among researchers on the symptoms of PSU, and some symptoms advocated by other researchers were not included in our network (46, 47). Besides, only one PSU scale was used in this study, and core symptoms from one scale may be highly associated due to methodological effects. Future studies could use multiple scales or different scales to explore core symptoms and their dynamic features. Second, the stability of the network analysis approach has been argued by several researchers (48, 49), which reminds us to interpret our results with caution. Third, the relatively long intervals between measurements may have led to sparse bidirectional prediction between symptoms and a weak predictive relationship from loss of control to being caught in the loop in the temporal network, and future studies could consider the intensive longitudinal design. Fourth, the data collection of wave 2 was conducted during COVID-19, and the increase in PSU from wave 1 to wave 2 and the decrease from wave 2 to wave 3 might be affected by COVID-19. Researchers suggested that, during the COVID-19 outbreak, adolescents increase smartphone use to search for information related to COVID-19, seek social support, and as a coping strategy (9, 50). During COVID-19 recovery (wave 3), the lifting of the “flexible learning at home” may reduce the need for online social support and information seeking (9). However, researchers also observed individual differences (9), and that PSU may increase before COVID-19 and decrease during the COVID-19 outbreak (51). These are reminders to interpret our results with caution. Fifth, this longitudinal study used different measurement formats (e.g., paper-and-pencil response and electronic response) at different waves, and it is unclear whether this practice has any impact. Finally, this study only explored the dynamic aspect of PSU symptoms, and future studies could explore the characteristics of network structure over time.

## Conclusion

At the longitudinal between-subjects level, the core symptoms of PSU, indeed, play key dominant roles in the future development of PSU symptoms and the severity of PSU. After separating the between- and within-subjects effects, the within- and between-subjects networks differed significantly. In the temporal network, only being caught in the loop plays an important role in the development of PSU symptoms. This study not only confirms and extends the critical role of core symptoms in the dynamics of PSU symptoms and PSU itself but also highlights the temporal variation of PSU symptoms at the within-subject level. Interventions should not only consider the core symptoms of PSU but also individual- and group-level effects, and individualized intervention programs are needed in future.

## Data availability statement

The raw data supporting the conclusions of this article will be made available by the authors, without undue reservation.

## Ethics statement

The studies involving human participants were reviewed and approved by the State Key Laboratory of Cognitive Neuroscience and Learning, Beijing Normal University. Written informed consent to participate in this study was provided by the participants’ legal guardian/next of kin.

## Author contributions

SH: conception and design, collection, analysis and interpretation of data, drafting the manuscript, and revising it critically for important intellectual content. XL: revising the draft critically for important intellectual content and collection. YL, WW, JL, and DL: collection. XD: investigation, reviewing, and edition of the draft. HW: reviewing and edition of the draft. YW: revising the draft critically for important intellectual content and final approval of the version to be published. All authors contributed to the article and approved the submitted version.
